# Isolated cryptococcal osteomyelitis in the setting of immune reconstitution inflammatory syndrome

**DOI:** 10.1177/20499361241230149

**Published:** 2024-02-07

**Authors:** Pamela Horton Embrey, Alexandra Long, Rasha Alfattal, Suimin Qiu, Joseph Patrik Hornak

**Affiliations:** University of Texas Medical Branch at Galveston, 301 University Blvd., Galveston, TX 77555, USA; University of Texas Medical Branch at Galveston, Galveston, TX, USA; University of Texas Medical Branch at Galveston, Galveston, TX, USA; University of Texas Medical Branch at Galveston, Galveston, TX, USA; University of Texas Medical Branch at Galveston, 301 University Blvd., Galveston, TX 77555, USA

**Keywords:** case report, *Cryptococcus*, fluconazole, HIV/AIDS, immune reconstitution inflammatory syndrome

## Abstract

Cryptococcal infections, though rare, must be considered in all immunocompromised patients. Patients with HIV/AIDS on antiretrovirals may have a treatment course complicated by immune reconstitution inflammatory syndrome. Here we present a case of a 38-year-old woman with HIV/AIDS with knee pain who only began to experience severe pain after induction of antiretroviral therapy. She was found to have cryptococcal osteomyelitis without dissemination to the central nervous system, an unusual presentation for immunocompromised patients. She was treated with oral fluconazole with a resolution of symptoms. This case report suggests conservative management of isolated cryptococcal infection with fluconazole, regardless of immune status.

## Plain language summary

Conservative treatment of isolated cryptococcus infection in a patient with a weakened immune system

Cryptococcus neoformans is a fungus found in the soil which care rarely infect humans, especially those who have a weakened immune system, like those with HIV infection. The treatment of HIV in people with ‘secondary infections’, like cryptococcal infections, may cause patients to get worse before they get better as the immune system starts to function and attack the secondary infection. In this case report, we look at a patient who had untreated HIV who only began to develop symptoms of a secondary cryptococcal infection once treatment for HIV was started. However, because the cryptococcal infection was only in her bone and not throughout her body and nervous system, we were able to treat her with a conservative, oral regimen. In patients with severe cryptococcal infection or with infection of their nervous system, they often need to be treated with medications that can cause a lot of unwanted side effects. The key takeaway from this article is that conservative treatment of Cryptococcus may be effective, even in people with weakened immune systems, as long as the cryptococcal infection is isolated.

## Background

*Cryptococcus neoformans* is an opportunistic pathogen that causes cryptococcal meningitis and pneumonia in immunocompromised individuals, although it has also been known to infect immunocompetent hosts.^1^ Infection is acquired through inhalation of spores, which become trapped in the alveoli and may invade through the alveolar–capillary membrane to the bloodstream, allowing the pathogen to spread hematogenously throughout the body.^2^ In immunocompromised hosts, the fungus is more likely to seed in multiple foci – most commonly the central nervous system and soft tissues. Disseminated cryptococcus results in severe infection requiring potent antifungal treatment with amphotericin B (AmB).^3^

The infection may sometimes lie dormant and manifest as part of immune reconstitution inflammatory syndrome (IRIS) in immunocompromised patients initiated on antiretroviral therapy.^4^ Nonetheless, cryptococcal IRIS has also been reported in immunocompetent individuals.^5^ As the immune system strengthens in a patient with a preexisting opportunistic infection, inflammation and inflammatory cytokines ramp up and can cause devastating side effects. IRIS is most common in infections with *Mycobacterium tuberculosis*, *Mycobacterium avium* complex, cryptococcal meningitis, cytomegalovirus retinitis, hepatitis B or C virus, progressive multifocal leukoencephalopathy, Kaposi’s sarcoma, and cerebral toxoplasmosis.^6^ Like primary infection, IRIS-associated cryptococcus has been predominantly cited in the form of meningitis. However, a few cases of isolated cryptococcus osteomyelitis without further disseminated infection have been reported in immunocompetent individuals.^7^ Here we describe an unusual case of isolated cryptococcus osteomyelitis of the knee in an HIV patient following the initiation of antiretroviral therapy and successfully treated with oral fluconazole.

## Case report

A 38-year-old woman with a past medical history of uncontrolled HIV/AIDS presented to an ambulatory orthopedic clinic for several weeks of anterior right knee pain after a fall several weeks prior, requiring the use of a walker. She had been noncompliant with her antiretrovirals in the past but recently began Biktarvy. Her CD4+ cell count was unknown on presentation. The history of previous opportunistic infections or complications of HIV/AIDS was unknown.

On examination, the patient had exquisite tenderness to palpation of the anterior knee, although there was no palpable defect or erythema. The straight leg test was negative and she had no instability. She had no fevers, oral thrush, or rashes.

**Figure 1.**
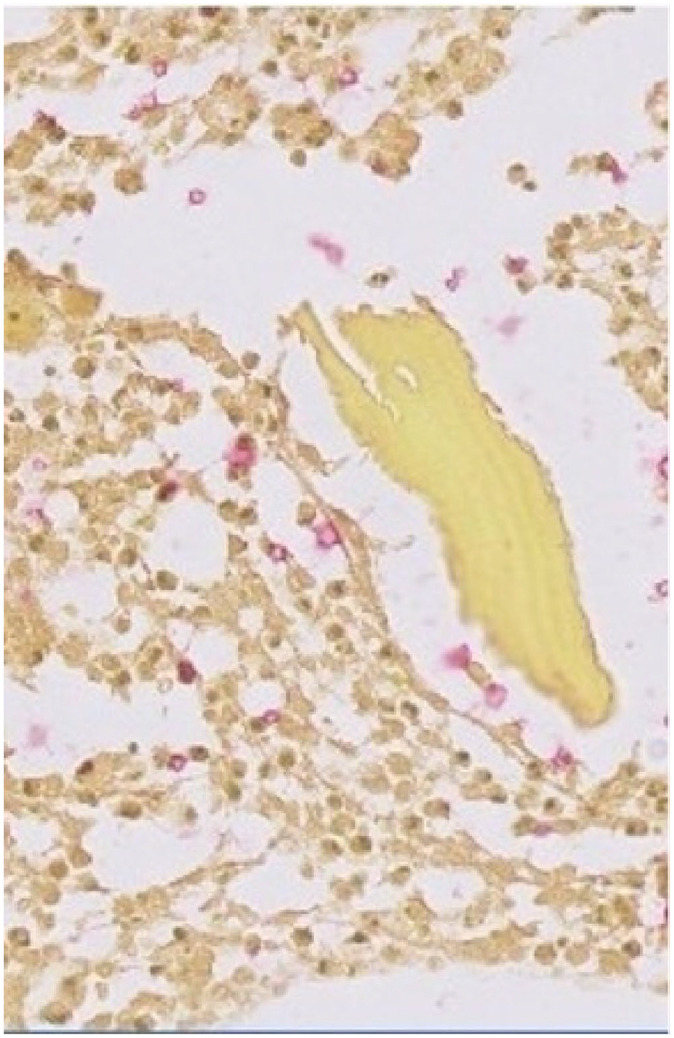
Mucicarmine staining using a Ventana staining kit of a bone fragment demonstrates the presence of a mucopolysaccharide capsule.

Initial workup revealed an erythrocyte sedimentation rate of 92 but no other significant symptoms. A diagnostic X-ray was ordered for a suspected patellar fracture but imaging instead showed patchy lucencies with soft tissue swelling. A follow-up computed tomography scan showed what appeared to be a well-circumscribed bone cyst measuring 1.5 cm × 1.2 cm. An arthroscopy was performed, which found a necrotic purulent anterior cortical erosion of the right patella. Empiric vancomycin/ceftriaxone therapy was started pending culture results. Pathology specimens of the resected bone revealed mixed acute and granulomatous inflammation with narrow-based budding yeasts. Grocott’s methenamine silver stain, mucicarmine staining (Figure 1), and cryptococcal antibody testing were positive confirming the diagnosis, and the patient was switched to oral fluconazole.

To characterize dissemination, serum and cerebrospinal fluid (CSF) antigen studies were performed. The serum was positive for cryptococcus by enzyme immunoassay with a titer of 1:96 but the CSF antigen was negative. To assess for pulmonary disease, a chest X-ray was performed, which was negative. Fungal cultures from the bone, blood, and CSF were negative.

During her hospital stay, the patient’s CD4+ count was found to be 104 by flow cytometry and her viral load was detectable, but unquantifiable. Based on the patient’s presentation coinciding with the recent resumption of HIV therapy, the patient was diagnosed with IRIS in association with a right patellar abscess and *C. neoformans* osteomyelitis. She was successfully discharged from the hospital after 2 weeks with instructions to continue oral fluconazole 200 mg once daily for at least 6 weeks.

## Discussion

Isolated cryptococcal osteomyelitis is a rare manifestation of *C. neoformans* infection and this may be the first reported case of IRIS-associated localized *C. neoformans* osteomyelitis.^8^ Current guidelines on the treatment of non-meningeal, non-pulmonary cryptococcus infection are based on limited evidence. There have been reports of cryptococcus osteoarticular infection without dissemination being treated with oral fluconazole but these were only seen in organ-transplant recipients, not HIV-infected individuals.^9^ For nonmeningeal, non-pulmonary cryptococcosis in *immunocompetent* individuals, the recommended treatment is with oral fluconazole for 6–12 months but this evidence is based on low-quality evidence. The treatment for immunosuppressed individuals with nonmeningeal, non-pulmonary cryptococcus, where disseminated infection has been ruled out by blood and CSF fungal cultures, has not been established. Of note, the treatment for cryptococcal meningoencephalitis in HIV-infected individuals includes AmB deoxycholate or the liposomal or lipid complex formulation of AmB plus flucytosine for 2 weeks, then fluconazole for at least 8 weeks.^9^ AmB is often used in disseminated fungal infections but is well known for its long list of severe side effects, including liver dysfunction, blood dyscrasias, electrolyte disturbances, severe nausea, anaphylaxis, acute renal failure, and cardiac arrhythmias.^10^ The use of this drug must therefore be reserved for severe fungal infections where the benefit outweighs the risks. If treatment with an antifungal drug with a more favorable side effect profile is possible, then the use of AmB should be spared. Here, we present a case where conservative treatment with oral fluconazole was effective in eradicating disease without the need to resort to intensive therapy with AmB. By contrast, fluconazole has a more favorable side effect profile and is much more likely than AmB to cause anemia, hypokalemia, or thrombocytopenia.^10^

Guidelines on the treatment of cryptococcus-associated IRIS are vague and limited to pulmonary or meningeal cryptococcus.^9,11^ It should be noted that in patients with HIV and cryptococcal meningoencephalitis, it is recommended to treat the cryptococcus for at least 2 weeks prior to initiating antiretrovirals to avoid IRIS.^12^ In pulmonary cryptococcus, it is recommended to continue fluconazole for 6–12 months based on B-III quality evidence. For cerebral cryptococcus infection, the recommendation is to continue induction therapy with AmB in immunocompromised patients as well as immune reconstituting drugs, again based on B-III evidence. No guidance is suggested for the management of isolated cryptococcal osteomyelitis in the setting of IRIS.

In this patient, there was a positive serum cryptococcal antigen but negative CSF antigen and cultures, allowing us to rule out meningeal or cerebral involvement. In addition, her symptoms only appeared once she had been on antiretrovirals for several weeks. It may be that she had an occult cryptococcus infection acquired either from the fall itself or from inhalation and hematogenous spread to the injured knee but had no symptoms until her immune system began to recover as an IRIS reaction. As we were unable to measure CD4+ count prior to initiation of Biktarvy, it is unclear how quickly her cell counts rose or how severe her immunodeficiency was initially.

## Conclusion

Our findings support individualization of treatment in cryptococcal infection where conservative treatment may be effective regardless of comorbidities such as HIV and IRIS. While guidelines may suggest the use of AmB in cryptococcosis in immunocompromised individuals, conservative therapy may be sufficient and spare patients from severe side effects. We also recommend the continuation of conservative therapy despite mild-to-moderate IRIS symptoms. Duration of therapy may differ between patients based on the clinical picture but patients with HIV, especially those with progression to AIDS, should be encouraged to continue antiretroviral therapy throughout and beyond the treatment period.

## Declarations

**Ethics approval and consent to participate:** Not applicable.

**Consent for publication:** The patient provided verbal and written informed consent for publication of this material.

**Availability of data and materials:** Not applicable. No new data were created in the preparation of this report. This case study complies with the CARE case report guidelines.
